# Reduced Gamma Oscillations in a Mouse Model of Intellectual Disability: A Role for Impaired Repetitive Neurotransmission?

**DOI:** 10.1371/journal.pone.0095871

**Published:** 2014-05-06

**Authors:** Andrew D. Powell, Pierre-Philippe Saintot, Kalbinder K. Gill, Ashtami Bharathan, S. Caroline Buck, Gareth Morris, Premysl Jiruska, John G. R. Jefferys

**Affiliations:** 1 School of Clinical and Experimental Medicine, College of Medical and Dental Sciences, University of Birmingham, Birmingham, United Kingdom; 2 Department of Developmental Epileptology, Institute of Physiology, Academy of Sciences of Czech Republic, Prague, Czech Republic; 3 Department of Neurology, Charles University, 2^nd^ School of Medicine, Prague, Czech Republic; Centre national de la recherche scientifique, University of Bordeaux, France

## Abstract

Intellectual disability affects 2–3% of the population; mutations of the X-chromosome are a major cause of moderate to severe cases. The link between the molecular consequences of the mutation and impaired cognitive function remains unclear. Loss of function mutations of oligophrenin-1 (*OPHN1*) disrupt Rho-GTPase signalling. Here we demonstrate abnormal neurotransmission at CA3 synapses in hippocampal slices from *Ophn1* ^-/y^ mice, resulting from a substantial decrease in the readily releasable pool of vesicles. As a result, synaptic transmission fails at high frequencies required for oscillations associated with cognitive functions. Both spontaneous and KA-induced gamma oscillations were reduced in *Ophn1* ^-/y^ hippocampal slices. Spontaneous oscillations were rapidly rescued by inhibition of the downstream signalling pathway of oligophrenin-1. These findings suggest that the intellectual disability due to mutations of oligophrenin-1 results from a synaptopathy and consequent network malfunction, providing a plausible mechanism for the learning disabilities. Furthermore, they raise the prospect of drug treatments for affected individuals.

## Introduction

The X-linked mental retardation (XLMR) disorders are a group of single gene mutations which lead to moderate to severe, non-specific, intellectual disability [1]. An ongoing major challenge is to understand the cellular mechanisms responsible for the cognitive impairment. Recent work has challenged the dogma that dendritic spine abnormalities are responsible for the underlying pathophysiology. It has led to the hypothesis that intellectual disabilities may be described as synaptopathies because several have been associated with alterations in synaptic function [2]–[4]. How these alterations in synaptic function affect brain function in ways that explain cognitive impairment remains unknown.

Synchronisation of neuronal activity in the gamma (30–80 Hz) frequency band is thought to underlie the encoding and retrieval of episodic memory, attention and the formation of neuronal assemblies that facilitate associative learning [5], [6]. Gamma oscillations have also been proposed to provide the spike timing that facilitates the synaptic summation necessary for long-term potentiation [7], [8]. Gamma oscillations are frequently studied in the hippocampus, where they have been suggested to function in exploratory behaviour and navigation [9]–[11] and gamma oscillation strength is correlated with behavioural performance [12]. Altered neuronal synchrony in the gamma band has been reported for a variety of neuropsychiatric disorders [5] and neurodevelopmental disorders, including autism, Williams syndrome [13] and the Ts65Dn Down syndrome model of intellectual disability [14].

Gamma oscillations can be generated *in vitro* in the CA3 region of the hippocampus by tonic activation of kainate (KA) receptors [15] or can occur spontaneously [16], [17]. Gamma and other cortical oscillations associated with cognition depend on the repetitive activity of inhibitory neurons and intermittent activity in excitatory neurons, and so the ability of inhibitory synapses to operate at these frequencies is critical [6], [18].

Loss of function mutations in the *OPHN1* gene (*Ophn1* in mice) result in a moderate to severe learning disability in humans [19] and learning impairments in mice [20]. *OPHN1* encodes oligophrenin-1, a protein with a Rho-GAP domain which negatively regulates RhoA, Rac1 and Cdc42 [19], [21]. In addition to the regulation of Rho-family GTPases, oligophrenin-1 regulates the size of the readily releasable pool (RRP) of vesicles in inhibitory synapses [4], possibly through regulation of synaptic vesicle endocytosis [22], [23]. The altered vesicle dynamics prevents synapses from functioning at frequencies within the gamma range [4].

In the present study, we investigate the role of oligophrenin-1 in neuronal network activity, particularly spontaneous and KA-induced gamma oscillations using the *Ophn1* mouse model of intellectual disability. We demonstrate that hippocampal synapses are unable to function at frequencies necessary for higher cognitive function, due to a substantial decrease in the RRP of synaptic vesicles. We propose that these synaptic changes underlie the deficits in gamma oscillations reported here. The alterations in spontaneous gamma oscillations were abolished by inhibition of the RhoA signalling pathway. The rescue of emergent neuronal network activity by small molecule pharmacological inhibition of the downstream signalling pathway of oligophrenin-1 raises the possibility of a pharmacotherapy to treat affected individuals.

## Methods

### Ethics statement

The *Ophn1* colony was generated using a C57-BL6 background as described previously [20]. *Ophn1* ^-/y^ mice and wild type (*Ophn1* ^+/y^) littermates were generated by breeding heterozygote females (*Ophn1* ^+/−^) with *Ophn1* ^+/y^ males; resulting in ∼50∶50 *Ophn1* ^+/y^:*Ophn1* ^−/y^. Breeding and experiments were performed under regulation by the Animals (Scientific Procedures) Act (1986) of the UK, and approved by the Biomedical Ethics Review sub-committee (BERSC) at the University of Birmingham. All experiments and analyses were performed blind to genotype.

### Hippocampal slice preparation

Horizontal hippocampal slices (400 µm for extracellular recordings, 250 µm for patch-clamp recordings) were prepared from *Ophn1* ^+/y^ and *Ophn1* ^−/y^ age-matched mice (3–9 weeks old) anaesthetised by intraperitoneal injection of medetomidine (1 mg/kg) and ketamine (76 /kg). Animals were transcardially perfused with ∼10 ml of ice-cold cutting solution comprising (mM): 189 sucrose, 26 NaHCO_3_, 1.2 NaH_2_PO_4_, 2.5 KCl, 0.1 CaCl_2_, 5 MgCl_2_ and 10 glucose (flow rate ∼2.7 ml/min). Slices were cut using an Integraslice (Campden Instruments, Loughborough, UK) and stored at room temperature in an interface chamber containing 95%O_2_–5%CO_2_ oxygenated artificial cerebrospinal fluid (aCSF)(in mM: 135 NaCl, 16 NaHCO_3_, 1.25 NaH_2_PO_4_, 3 KCl, 2 CaCl_2_, 1 MgCl_2_ and 10 glucose, pH 7.4).

### Extracellular recordings

After sectioning, slices were placed in a Haas-type interface recording chamber and allowed to equilibrate for an hour at the interface between aCSF and moist 95%O_2_–5%CO_2_ (300 cm^3^/min). Slices were constantly perfused with aCSF at a flow rate of ∼2 ml/min; the temperature was maintained at 32°C. Slices were visualised with a stereo-microscope (Leica MZ8, Micro Instruments, Long Hanborough, Oxon, UK) mounted above the interface chamber. Extracellular microelectrodes were pulled from thick-walled borosilicate glass capillaries (1.2 mm O.D.×0.69 mm I.D.; Harvard apparatus, Edenbridge, Kent, UK) using a P-97 puller (Sutter Instrument Co, Novato, CA). Electrodes were filled with aCSF and had a typical resistance of 2–4 MΩ. Extracellular potentials were recorded using an Axoclamp 2B amplifier (Molecular Devices, Sunnyvale, CA), low pass Bessel filtered at 1 kHz (NL-125, Digitimer Ltd, Welwyn Garden City, UK) and digitized at 10 kHz by a Power 1401 (CED Ltd, Cambridge, UK). Additionally, a Humbug 50/60 Hz (Digitimer) was used to remove noise locked to the electrical mains supply. Stimulation and data acquisition were controlled using Spike 2 software (v6.12; CED). Data were stored for subsequent off-line analysis using Spike 2.

### Gamma oscillations

After slices were placed in the interface recording chamber, an extracellular recording electrode was placed in the pyramidal cell layer of CA3c. Spontaneous activity was recorded for 5 minutes before the addition of 50 nM KA. The strength of the gamma oscillation in the frequency range 20–80 Hz was measured by fast Fourier transforms of 60 s epochs of data (Hanning window, FFT size 4096) and quantified as summated power. The dominant frequency in this range was used to quantify the peak frequency. Spontaneous gamma oscillations were observed in a subset of slices (*Ophn1* ^+/y^ 8 of 21 slices [∼38%]; *Ophn1* ^−/y^ 10 of 30 slices [∼33%]), characterised by a peak power in the gamma range (average power in 25–35 Hz is greater than average power in 10–20 Hz) [16].

Waveform averages were calculated from 30–50 gamma cycles, phase zeroed at the peak of the gamma cycle recorded in s. pyramidale. To avoid potential bias between genotypes, the following method was used to select the gamma cycles for averaging. A 2 s epoch of data was band pass filtered (20–80 Hz, 2^nd^ order Butterworth digital filter) and events between 30 and 70% of the maximum filtered signal were selected for averaging.

The effect of Rho kinase (ROCK) inhibitors on gamma oscillations were evaluated by addition of Y–27632 (trans-4-[(1R)-1-Aminoethyl]-N-4-pyridinylcyclohexanecarboxamide dihydrochloride; 10 µM) to the superfusate for 20 minutes before the addition of 50 nM KA. The effect of Y–27632 on spontaneous gamma oscillations was tested immediately before addition of KA and on KA–induced gamma oscillations after 1 hour of KA.

### Evoked field potentials

The mossy fibre tract was stimulated using a nichrome concentric stimulating electrode (80% nickel/20% chromium, tip diameter 50 µm; Advent research materials; Oxford, UK) placed in the hilus. Postsynaptic potentials (PSPs) were recorded from s. radiatum in CA3c. Stimulus-response curves were generated to determine the half maximal stimulus intensity values (20.8±0.6 V, range 15–27 V, n = 31). This intensity was used for subsequent high frequency stimulation recordings. High frequency stimulation was evoked by a train of 10 stimuli at 33 Hz and PSP slopes were measured and normalised to the first response.

### Whole-cell patch-clamp recordings

Whole-cell patch-clamp recordings were made from the somata of CA3c pyramidal neurons using infrared DIC (Olympus BX-51 upright microscope, fluorplan 40x, 0.8 NA water immersion lens; Micro Instruments, Long Hanborough, UK). Patch electrodes were pulled from borosilicate glass (O.D. 1.2 mm, I.D. 0.69 mm) using a P-97 puller. For IPSC studies, intracellular solution comprised (mM): 135 CsCl_2_, 2 MgCl_2_, 10 HEPES, 5 QX-314, 1 EGTA, 2 Mg-ATP and 0.3 Na-GTP; pH 7.3 with KOH (osmolarity ∼285 mOsm); this shifted the reversal potential to 0 mV, resulting in inward IPSCs at −70 mV. Synaptic currents using this electrode solution were confirmed as IPSCs by their abolition by 10 µM bicuculline (not shown). For EPSC studies, intracellular solution comprised (mM): 140 CsCH_3_SO_4_, 8 NaCl, 10 HEPES, 5 QX-314, 2 Mg-ATP and 0.3 Na-GTP; pH 7.3 with KOH (osmolarity ∼285 mOsm). EPSCs were recorded at −75 mV to avoid contamination by IPSCs (reversal potential of IPSCs) and were confirmed as EPSCs by their abolition by 20 µM NBQX and 25 µM d-APV (not shown). Patch electrodes typically had resistances of 4–7 MΩ. Membrane potentials and currents were recorded using an NPI SEC-10L amplifier (Scientifica, Harpenden, UK), low pass Bessel filtered at 1 kHz (NL-125, Digitimer Ltd, Welwyn Garden City, UK) and digitized at 10 kHz by a Power 1401 (CED Ltd, Cambridge, UK). Stimulation and data acquisition were controlled using Signal software (v3.10; CED). Evoked currents were elicited by a concentric stimulating electrode placed in the hilar region.

The readily releasable pool (mature and fusion-competent) was quantified by cumulative evoked IPSC amplitude analysis during repetitive stimulation [24]. The intracellular solution was supplemented with 100 µM Alexa 488 to enable visualisation of dendrites. A stimulating electrode (2^nd^ patch pipette filled with aCSF) was placed near the dendritic tree ∼50 µm from the soma. Stimulus intensity was varied to achieve minimal stimulation (activation of a single axon/synapse) as described in Powell *et al* (2012). The RRP size and probability of vesicle release from the RRP were calculated by applying repetitive stimuli (40 pulses at 20 Hz) and calculated as outlined in the main text.

### Statistical analysis

Values are expressed as mean±s.e.m. Curve fitting and data analysis were performed in Origin 8 (Silverdale Scientific, Stoke Mandeville, UK). Unpaired *t*-tests (with Bonferonni correction for multiple comparisons) were used to evaluate differences between genotypes; a Shapiro-Wilk test was used to examine if the data followed a normal distribution. Repetitive stimulation experiments were analysed using ANOVA. Significance criterion was *p*<0.05.

### Drugs

All chemicals were purchased from Abcam Biochemicals (Cambridge, UK).

## Results

**Figure 1:**
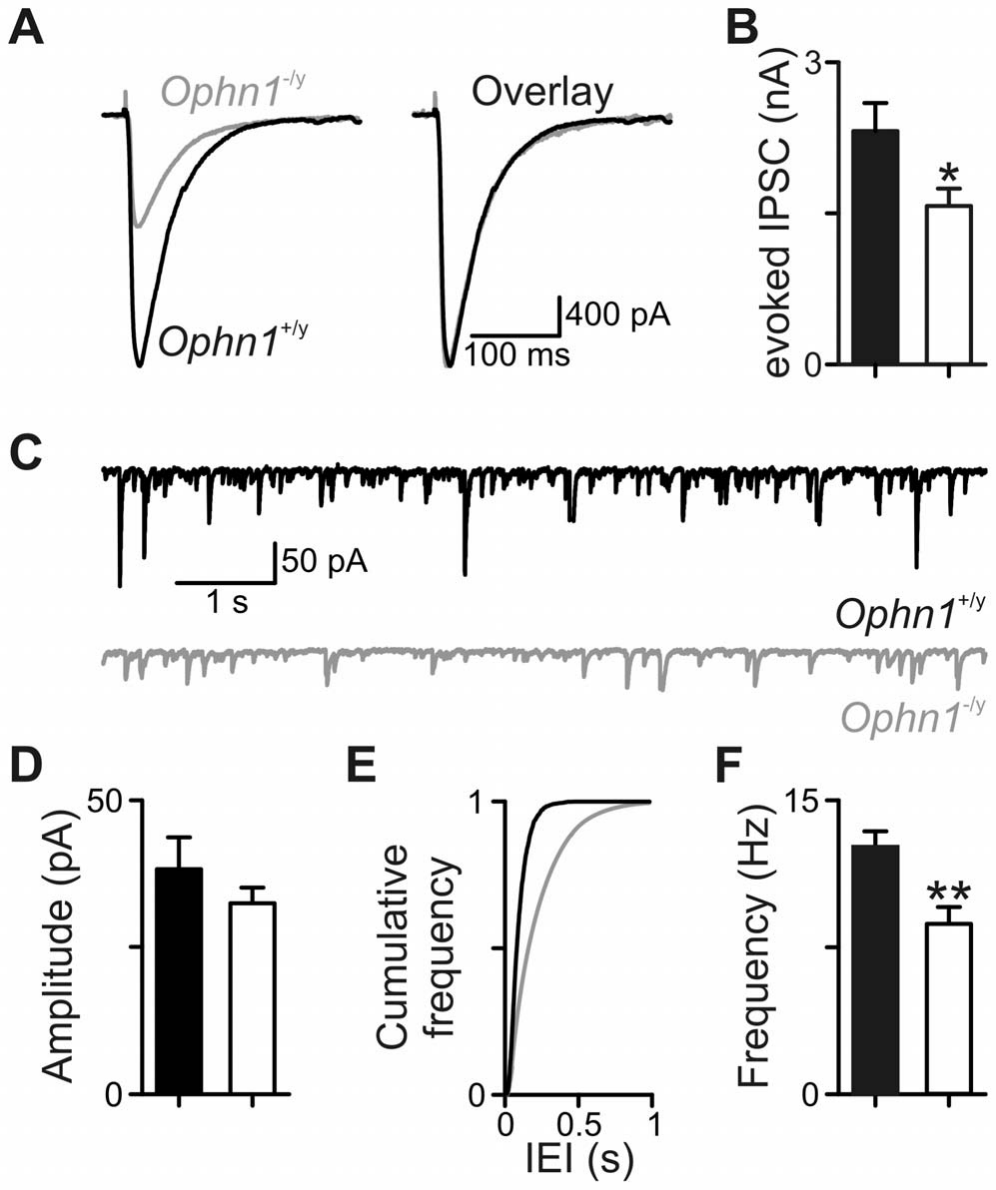
Inhibitory transmission is reduced in CA3 synapses. (**a**) Evoked IPSCs were smaller in *Ophn1* ^−/y^ (*grey trace*) than *Ophn1* ^+/y^ (*black trace*) neurons. Normalisation (*right panel*) of the *Ophn1* ^−/y^ evoked IPSC (*grey trace*) revealed that the kinetics of the eIPSCs were unaltered by genotype. (**b**) Mean evoked IPSC amplitude for an 18 V stimulus applied to mossy fibre pathway. (**c**) Spontaneous IPSCs were less frequent in *Ophn1* ^−/y^ (*grey trace*) than *Ophn1* ^+/y^ (*black trace*) neurons. Cumulative frequency plots (**e**) showed that spontaneous events in *Ophn1* ^−/y^ neurons shifted to longer inter-event intervals (IEI, *grey line*), which resulted in a lower frequency of spontaneous IPSCs (**f**). (**d**) The amplitude of spontaneous IPSCs was unaltered. (*p*:*<0.05, **<0.01). *Ophn1* ^+/y^ - filled columns, *Ophn1* ^−/y^.

We have previously demonstrated that repetitive inhibitory neurotransmission onto dentate gyrus granule neurons is reduced due to a smaller RRP [4]. We first tested whether the central conclusions from dentate granule cells generalised to CA3 pyramidal neurons. IPSCs evoked by stimulating the mossy fibre inputs were significantly smaller in *Ophn1* ^−/y^ neurons than *Ophn1* ^+/y^ neurons (1573±173 pA, n = 15 and 2317±279 pA, n = 13, respectively; *p* = 0.02; Fig 1a, b); neither the slope (*Ophn1* ^−/y^ 0.13±0.02 pA/V, *Ophn1* ^+/y^ 0.15±0.03 pA/V, *p* = 0.64), nor the half maximal stimulus strength (*Ophn1* ^−/y^ 10.6±1.4 V, *Ophn1* ^+/y^ 8.2±1.0 V, *p* = 0.2) of the stimulus response curve were altered in *Ophn1* ^−/y^ neurons. The frequency of spontaneous IPSCs was lower in *Ophn1* ^−/y^ neurons than *Ophn1* ^+/y^ neurons (8.8±0.8 Hz, n = 7 and 12.6±0.8 Hz, n = 7, respectively; *p* = 0.009; Fig 1c, e, f), consistent with the smaller evoked IPSCs. The amplitude of spontaneous IPSCs was unaffected in *Ophn1* ^−/y^ neurons (*Ophn1* ^−/y^ 32.5±2.7 pA, n = 7, *Ophn1* ^+/y^ 38.3±5.4 pA, n = 7; *p* = 0.35; Fig 1d).

### *Ophn1* null neurons display altered responses to repetitive stimuli

**Figure 2:**
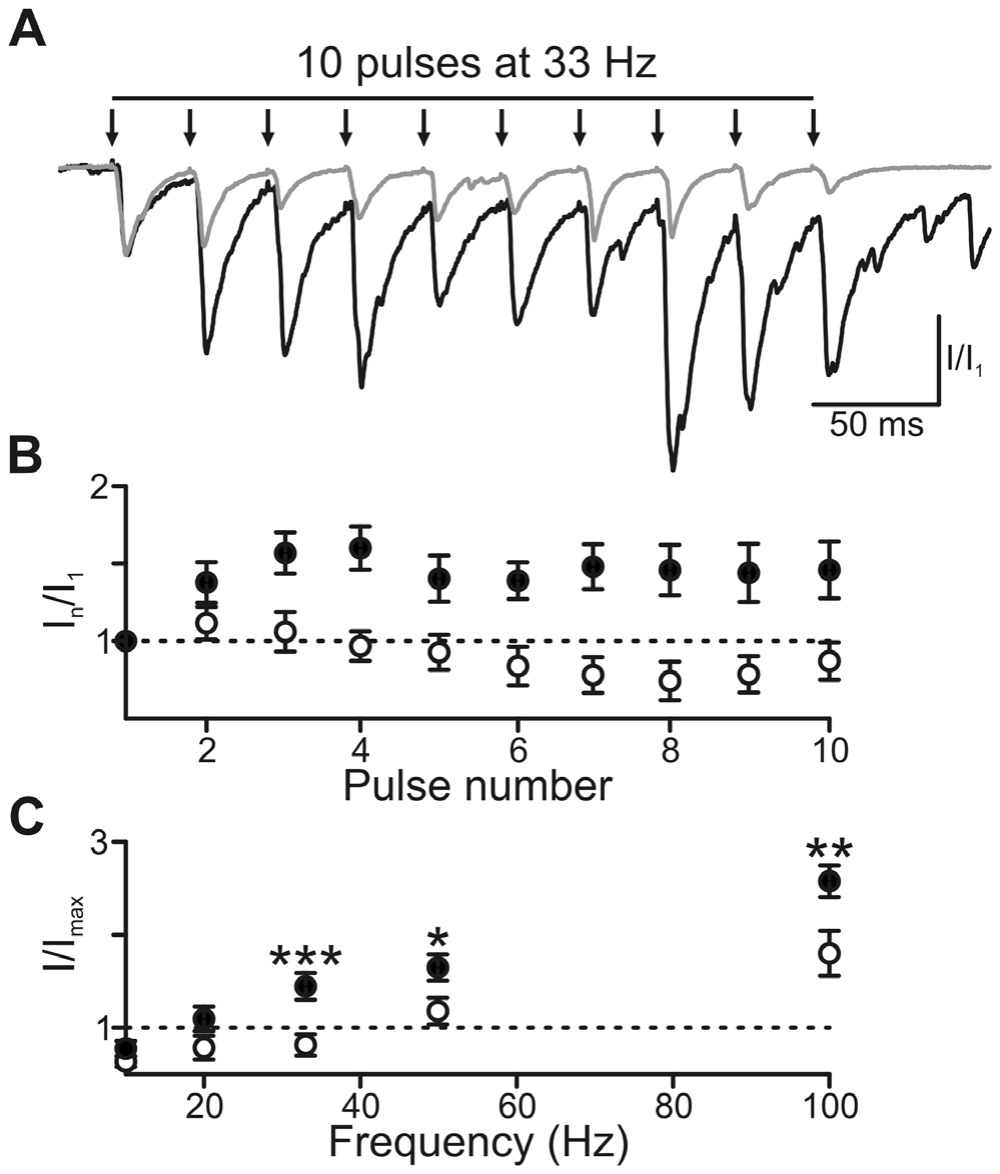
Reduced facilitation in response to high frequency stimulation in *Ophn1* ^−/y^ neurons. (**a**) Representative traces illustrating IPSC summation in response to 10 stimuli delivered at 33 Hz. The responses to first stimuli were normalised. In contrast to *Ophn1* ^+/y^ neurons, *Ophn1* ^−/y^ IPSCs (*grey trace*) showed no summation. (**b**) IPSC amplitude plotted against stimulus number for 33 Hz trains in *Ophn1* ^+/y^ (•, n = 20) and *Ophn1* ^−/y^ neurons (○, n = 14). (**c**) Maximal IPSC amplitude (mean of the last 5 stimuli) plotted against stimulus frequency. (*p*: *<0.05, **<0.01, ***<0.001).

**Figure 3:**
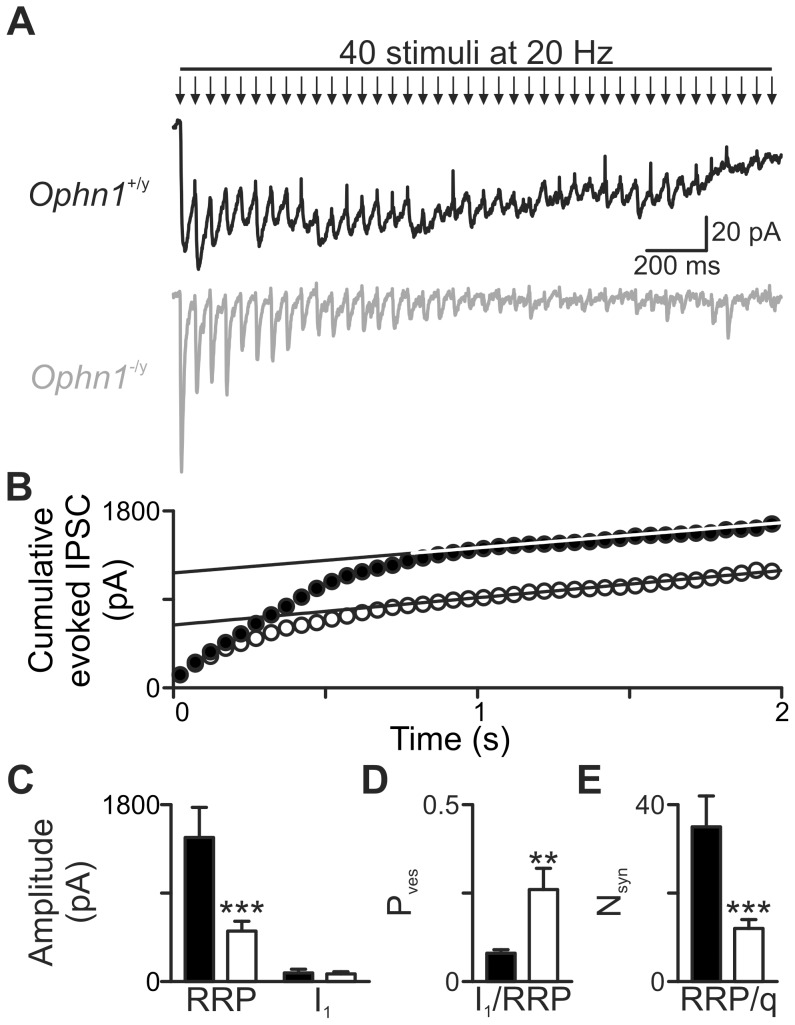
RRP is reduced in CA3 synapses. Evoked IPSCs recorded during a 20*Ophn1* ^+/y^ (**a**, *black trace*) and *Ophn1* ^−/y^ neuron (*grey trace*). The traces are averages of 5 sweeps. (**b**) The corresponding cumulative evoked IPSC amplitude plot (*Ophn1^+/y^*,•; *Ophn1* ^−/y^,○). Data between 1–2 s were fitted by linear regression and back-extrapolated to time 0 to estimate the RRP size (**b, c**). (**c**) The mean amplitude of IPSC_1_ was unaltered in *Ophn1* ^−/y^ neurons. (**d**) Mean P_ves_ was increased in *Ophn1* ^−/y^ neurons, whilst the mean number of vesicles (N_syn_) forming the RRP was reduced (**e**) (*p*: **<0.01, ***<0.005).

We examined the ability of synapses to follow high frequency stimulation [25] at frequencies associated with cognition (≥33 Hz) [6]. Using a submaximal stimulus (evoked ∼30% of maximum evoked IPSC), IPSCs built up with successive stimuli at 33 Hz, reaching a steady level within 10 stimuli in *Ophn1* ^+/y^ neurons (144±14%, n = 14; Fig 2a, b). IPSC facilitation did not occur in *Ophn1* ^−/y^ neurons (82±12%, n = 20; *p* = 0.003; Fig 2c). Facilitation was also weakened in *Ophn1* ^−/y^ neurons for 50 and 100 Hz stimulation (Fig 2c). As oligophrenin-1 controls the size of the RRP [4], we reasoned that this may explain the altered response to repetitive stimuli in *Ophn1* ^−/y^ CA3 neurons. Using high frequency minimal stimulation (40 pulses, 20 Hz) [4], [24], we examined the functional state of the RRP in CA3 neurons. The RRP was significantly smaller in *Ophn1* ^−/y^ neurons than in *Ophn1* ^+/y^ neurons (513.8±100.9 pA, and 1466.2±307.7, respectively, n = 7; *p* = 0.005; Fig 3b, c), corresponding to a smaller number of vesicles in the RRP (12±2 and 34±7, respectively; *p* = 0.005; Fig 3e). The reduction in RRP did not alter the mean amplitude of the first IPSC (*Ophn1* ^−/y^ 97.8±19.9 pA and *Ophn1* ^+/y^ 75.4±16.3 pA, *p* = 0.84; Fig 3c), which means that the probability of releasing single vesicles from the RRP (P_ves_) was significantly greater in *Ophn1* ^−/y^ neurons (*Ophn1* ^−/y^ 0.26±0.06 and *Ophn1* ^+/y^ 0.08±0.01, *p*<0.01; Fig 3d).

### Altered responses to repetitive stimuli are rescued by ROCK inhibition

**Figure 4:**
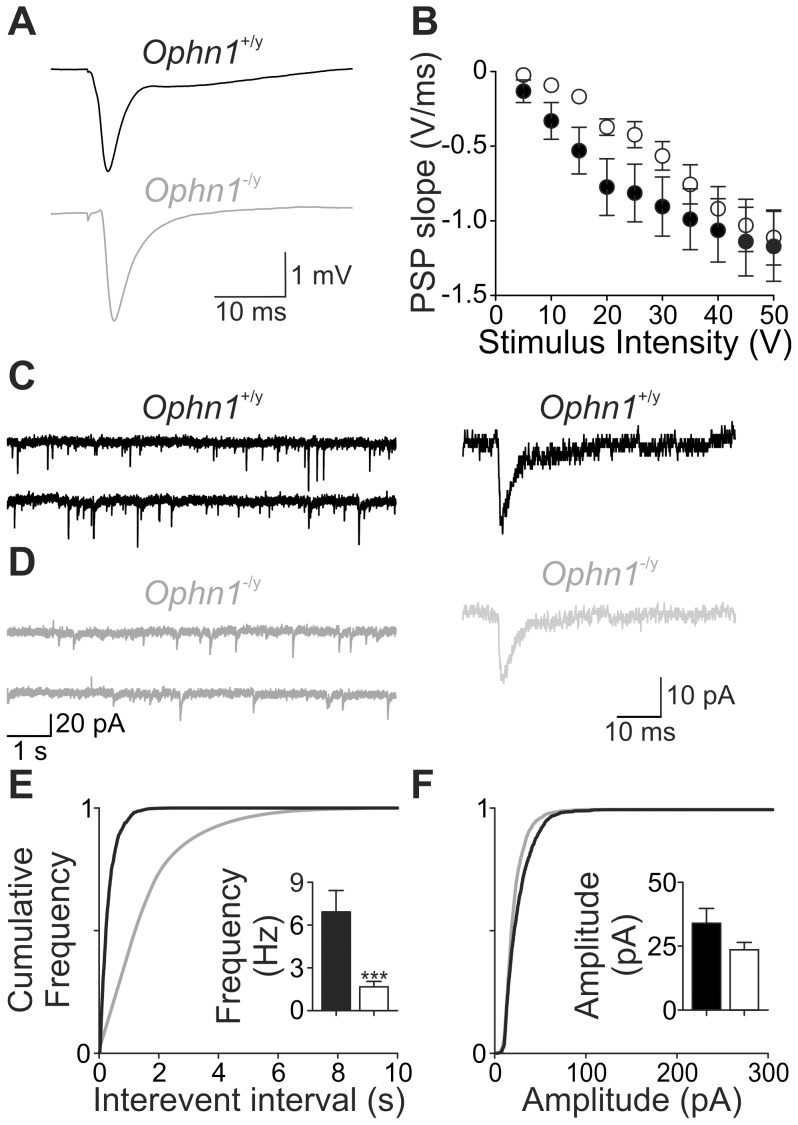
*Ophn1* ^−/y^ slices show reduced postsynaptic potentials. (**a**) Representative traces of postsynaptic potentials from *Ophn1* ^+/y^ (black traces) and *Ophn1* ^−/y^ (grey traces) slices. (**b**) Stimulus response curve of postsynaptic potentials recorded from the s. radiatum of CA3. PSP slopes were significantly smaller in *Ophn1* ^−/y^ (n = 5) than in *Ophn1* ^+/y^ slices (n = 12; p = 0.011, ANOVA). Representative traces of spontaneous EPSCs in *Ophn1* ^+/y^ (**c**) and *Ophn1* ^−/y^ (**d**). Representative individual spontaneous EPSCs are shown in the right panel. Cumulative frequency plots show that the interevent intervals (**e**), but not amplitude (**f**) of EPSCs are altered in *Ophn1^−^* ^/y^ neurons compared to *Ophn1* ^+/y^ neurons. (*inset*) Mean frequency and amplitude of spontaneous EPSCs.

Having demonstrated that inhibitory neurotransmission in the CA3 region is affected in a manner similar to that in the dentate gyrus [4], we examined whether these observations translated to excitatory transmission. Extracellular field potentials were recorded from CA3 s. radiatum and PSPs were evoked by activation of the mossy fibre pathway. PSPs were reduced in *Ophn1* ^−/y^ slices (Fig 4a, B; p<0.01) and the frequency of spontaneous excitatory postsynaptic currents recorded from CA3 pyramidal neurons was lower in *Ophn1* ^−/y^ neurons than *Ophn1* ^+/y^ neurons (1.7±0.4 Hz, n = 9 and 6.9±1.5 Hz, n = 8, respectively; *p* = 0.003; Fig4 c–f).

**Figure 5:**
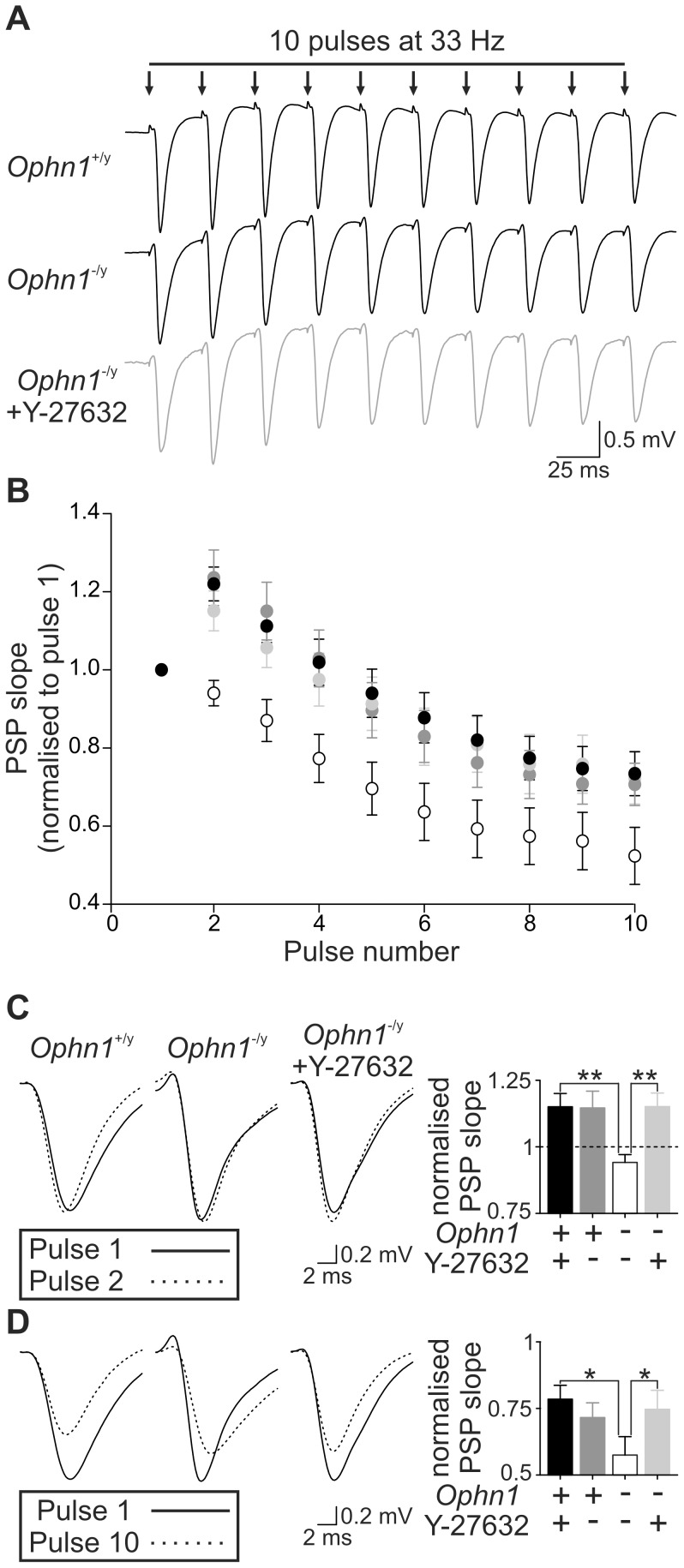
Reduced synaptic responses to high frequency stimulation in *Ophn1* ^−/y^ slices are rescued by ROCK inhibition. (**a**) Representative traces illustrating PSP recordings from CA3b s. radiatum in response to 10 stimuli delivered at 33 Hz. The responses to first stimuli were normalised. In contrast to *Ophn1* ^+/y^ neurons, *Ophn1* ^−/y^ PSPs showed no potentiation, but were rescued by superfusion with Y-27632 (10 µM; 20 minutes). (**b**) PSP slope plotted against stimulus number for 33 Hz trains in *Ophn1* ^+/y^ slices under control conditions (*black symbols*) and 20 minutes after Y-27632 application (*dark grey symbols*, n = 11). The reduced facilitation in *Ophn1* ^−/y^ slices (*open symbols*) was rescued by 20 minute application of Y-27632 (*light grey symbols*, n = 11). (**c**) Potentiation of PSP evoked by the 2^nd^ stimuli was reduced in *Ophn1* ^−/y^ slices and rescued by Y-27632 (*right panel*). (**d**) Synaptic depression (average PSP for pulses 8–10) was enhanced in *Ophn1* ^−/y^ slices and rescued by Y-27632 (*right panel*).

To test this deficit further, we examined how excitatory synapses responded to stimulation at frequencies associated with cognition. In response to a train of stimuli (10 at 33 Hz), synaptic potentials from *Ophn1* ^+/y^ slices initially showed potentiation (pulse 2, 1.15±0.05) followed by depression (pulses 8–10, 0.78±0.05; Fig 5b). In contrast, *Ophn1* ^−/y^ responses did not show potentiation (pulse 2, 0.94±0.03; p = 0.002) and the depression observed with later stimuli was more marked (pulses 8–10, 0.57±0.7; p = 0.03).

We have previously demonstrated that altered synaptic physiology observed in the absence of oligophrenin-1 can be reversed by acute inhibition of ROCK [4]. Inhibition of ROCK by Y-27632 (10 µM, 20 minutes) did not affect responses in *Ophn1* ^+/y^ slices (pulse 2, 1.15±0.06; p = 0.91, pulses 8–10, 0.72±0.06; p = 0.76). In contrast, *Ophn1* ^−/y^ responses were restored to similar levels as *Ophn1* ^+/y^ slices (pulse 2, 1.15±0.05; p = 0.001, pulses 8–10, 0.75±0.07; p = 0.01).

### Altered gamma oscillations in *Ophn1^−/y^* slices

Synchronisation of neuronal activity in the gamma frequency range has been associated with cognitive function [6] and can be recorded *in vitro* either spontaneously [16] or generated by superfusion of KA [6].

**Figure 6:**
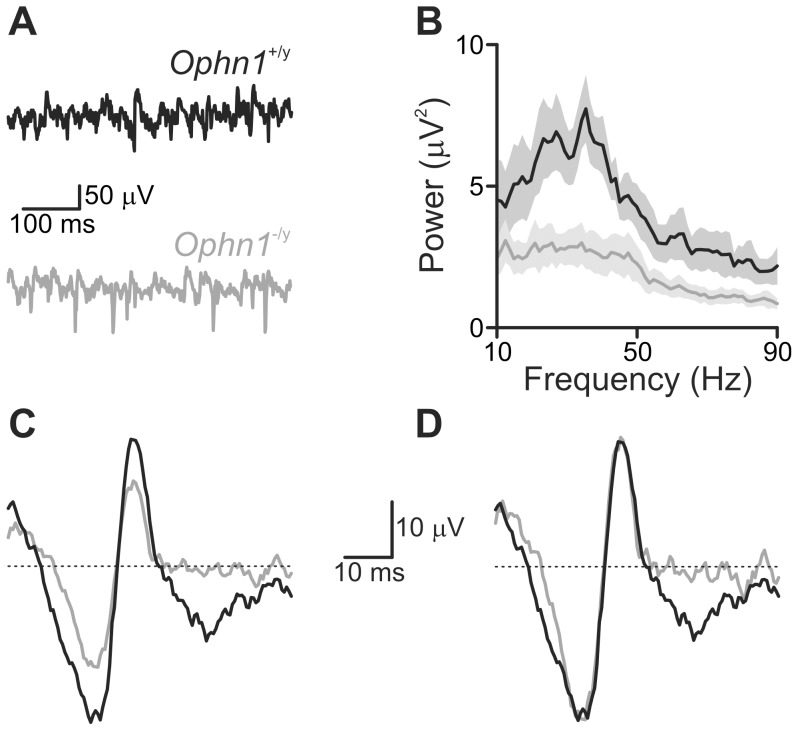
Spontaneous gamma oscillations are reduced in *Ophn1* ^−/y^ slices. (**a**) Representative traces from *Ophn1* ^+/y^ (*black traces*) and *Ophn1* ^−/y^ (*grey traces*) slices. The power of spontaneous gamma oscillations was reduced in *Ophn1* ^−/y^ slices (**b**). Grey shading indicates s.e.m. Normalisation (**d**) of the *Ophn1* ^−/y^ average gamma waveform (**c**) revealed that the kinetics of the spontaneous gamma oscillations were unaltered by genotype.

Hippocampal slices were placed in the interface recording chamber and an extracellular recording electrode was placed in the pyramidal cell layer of CA3c. Spontaneous activity was recorded for 5 minutes before addition of KA (Fig 6a, 7a). The summated power of spontaneous gamma oscillations was smaller in *Ophn1* ^−/y^ slices than in *Ophn1* ^+/y^ slices (27.5±7.6 µV^2^, n = 8 and 66.0±13.7 µV^2^, n = 10, respectively, *p* = 0.03; Fig 6b). Calculation of the waveform average for spontaneous gamma oscillations in *Ophn1* ^+/y^ and *Ophn1* ^−/y^ slices revealed that, despite the reduction in overall gamma power, the shape of the gamma cycle was unaltered (Fig 6c, d).

**Figure 7:**
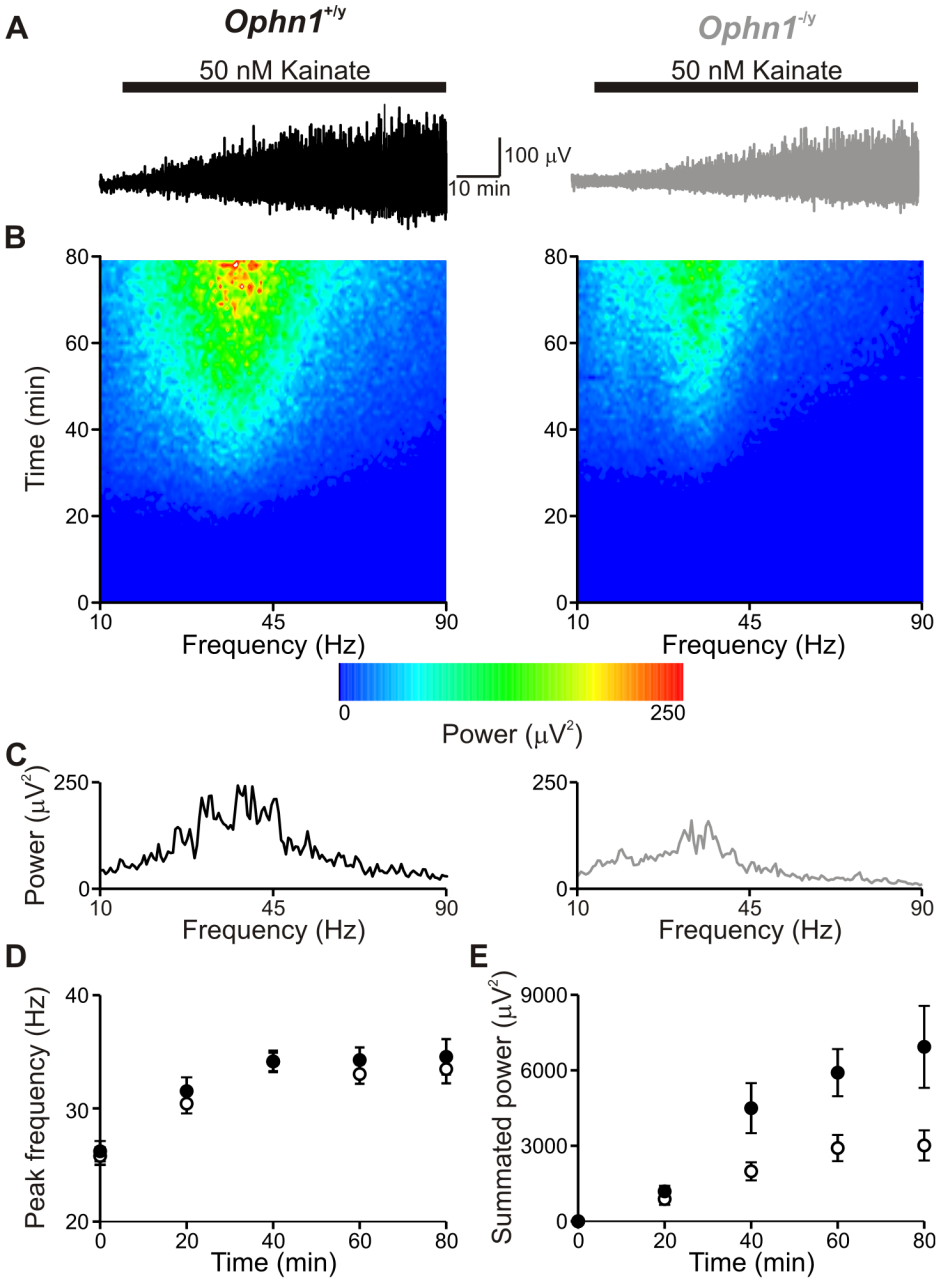
Smaller gamma oscillations in *Ophn1* ^−/y^ slices. (**a**) Application of 50 nM KA induced neuronal synchrony in the gamma frequency range; the power of these oscillations increased over time (**e**). (**b**) Spectrogram illustrating the development of the dominant frequency of gamma oscillations in *Ophn1* ^+/y^ (*left panel*) and *Ophn1* ^−/y^ (*right panel*) slices. (**c**) Power spectra for *Ophn1* ^+/y^ (*left panel*) and *Ophn1* ^−/y^ (*right panel*) slices at t = 60 minutes. (**d**) The peak frequency did not differ significantly between *Ophn1* ^+/y^ (•) and *Ophn1* ^−/y^ (○) slices. (**e**) Summated power of gamma oscillations was reduced in *Ophn1* ^−/y^ slices.

**Figure 8:**
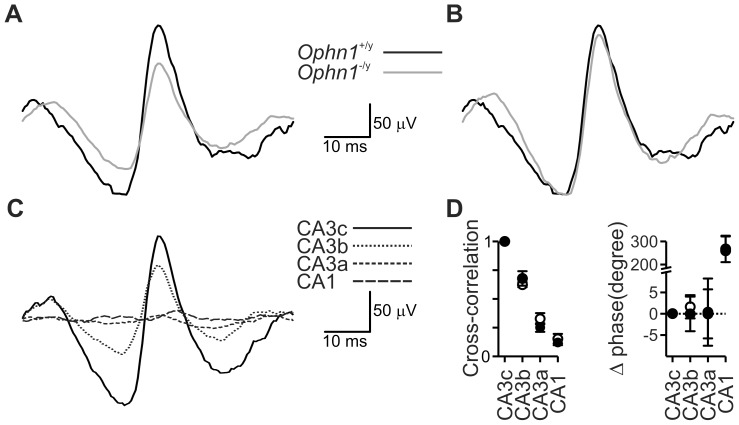
Gamma oscillation synchrony and coherence unchanged in *Ophn1* ^−/y^ slices. (**a**) Average gamma waveforms for *Ophn1* ^+/y^ (*black trace*) and *Ophn1* ^−/y^ (*grey trace*) slices revealed a reduced amplitude without alteration in gamma waveform kinetics (**b**; *grey trace*, normalised *Ophn1* ^−/y^). The reduced gamma power in *Ophn1* ^−/y^ slices was not associated with an altered spatial distribution; (**c**) waveform averages phase-zeroed at the peak of the oscillation recorded from CA3c (*black trace*), CA3b (*dotted trace*), CA3a (*short dashed trace*) and CA1 (*long dashed trace*) in an *Ophn1* ^+/y^ slice. (**d**) Cross-correlation (*left panel*) and phase lead (*right panel*) for CA regions with CA3c as the reference, data are expressed as mean±s.e.m. No differences were observed between *Ophn1* ^+/y^ (*filled symbols*) and *Ophn1* ^−/y^ (*open symbols*) slices.

Superfusion of KA (50 nM) increased CA3 network activity throughout its application in both *Ophn1* ^+/y^ and *Ophn1* ^−/y^ slices (Fig 7a, b). The frequency of oscillations increased in *Ophn1* ^−/y^ slices from 26.2±0.9 Hz for baseline activity to 34.3±1.1 Hz after 1 hr (n = 18; Fig 7d). A similar increase in frequency was observed in *Ophn1* ^+/y^ slices (spontaneous 25.8±0.8 Hz, to 33.0±0.9 Hz after 1 hr, n = 28; Fig 7d). KA increased the strength of oscillation in *Ophn1* ^+/y^ slices (20 minutes 1188.8±221.7 µV^2^, 60 minutes 5906.2±936.9, n = 30; Fig 7c). In *Ophn1* ^−/y^ slices, the increase in gamma power was significantly smaller throughout the superfusion of KA (20 minutes 882.8±230.5 µV^2^, 60 minutes 2910.1±521.3, n = 21; Fig 7c). Analysis of the representative spectrograms for the development of KA-induced oscillations did not reveal major differences between *Ophn1* ^+/y^ and *Ophn1* ^−/y^ slices (Fig 7b), indeed it appeared to be a simple loss of power because the average gamma waveform was unaltered (Fig 8b).

Given that *Ophn1* ^−/y^ mice show alterations in dendritic morphology [4], [26], we reasoned that associated changes in connectivity may affect the spatial distribution of KA-induced gamma oscillations. To compare the spatial distribution and coherence of KA-induced gamma oscillations, field recordings were made along the pyramidal layer of the CA regions. A stationary electrode was placed in CA3c and a roving electrode was placed in CA3b, CA3a and CA1 in turn. The maximum cross-correlation and phase difference were determined from cross-correlograms using CA3c as the reference channel. As shown previously [16], KA-induced gamma oscillations were coherent throughout the CA regions, but the maximum cross-correlation fell with increasing electrode separation. No significant differences between *Ophn1* ^+/y^ and *Ophn1* ^−/y^ slices were observed for spatial profile or coherence (Fig 8c, d).

### Sensitivity of reduced gamma oscillations to ROCK inhibitors

**Figure 9:**
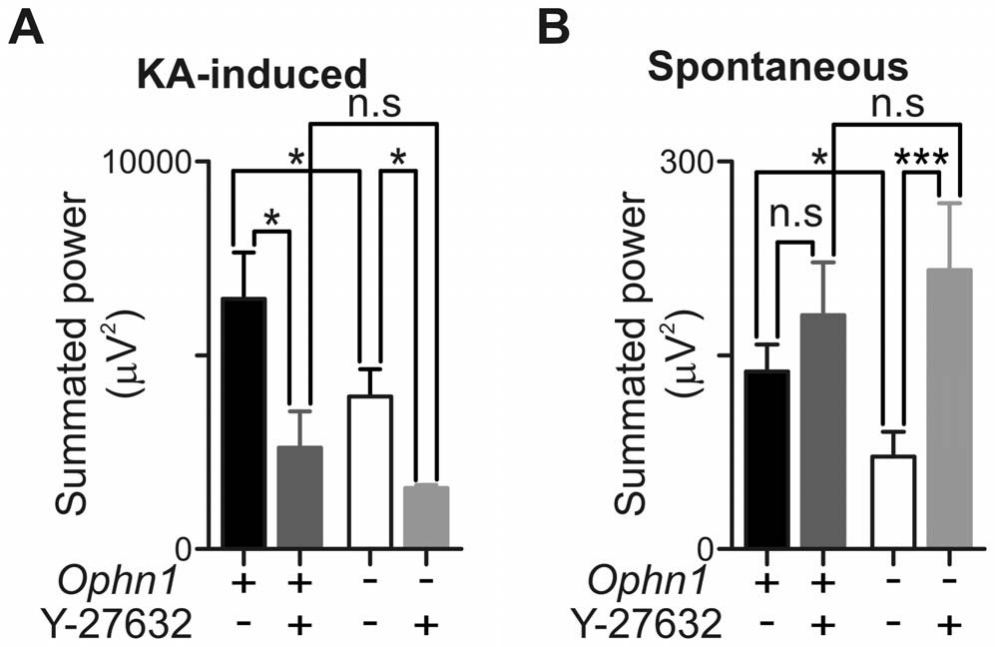
KA-induced and spontaneous gamma oscillations are differentially affected by ROCK inhibition. Y–27632 (10 µM) was applied for 20 minutes prior to application of KA (50 nM). (**a**) Oscillations recorded at 60 minutes post KA application were reduced by Y-27632 application. In contrast, spontaneous oscillations recorded prior to KA application were enhanced in *Ophn1* ^−/y^ slices (**b**) (*p*: *<0.05, ***<0.005).

As the synaptic deficits in *Ophn1* ^−/y^ dentate gyrus granule cells are reversed by acute inhibition of ROCK activity [4], we examined whether the deficit in network oscillations could also be reversed. Application of Y–27632 (10 µM) for 20 minutes prior to superfusion of KA did not reverse the impairment in *Ophn1* ^−/y^ slices, but rather depressed KA-induced gamma oscillations in both *Ophn1* ^+/y^ and *Ophn1* ^−/y^ slices. After 60 minutes of KA, Y–27632 had reduced gamma oscillations in *Ophn1* ^+/y^ slices to ∼50% of those in vehicle controls (2315.3±1056.6 µV^2^, n = 7 and 6146.8±1317.9 µV^2^, n = 10, respectively, *p* = 0.04; Fig 9a). Application of Y–27632 to *Ophn1* ^−/y^ slices caused a similar reduction in KA-induced gamma oscillations from vehicle controls (1272.8±197.3 µV^2^, n = 5 and 3638.0±823.2 µV^2^, n = 11, respectively, *p* = 0.02; Fig 9a).

Inhibition of ROCK by Y-27632 in *Ophn1* ^−/y^ slices significantly increased spontaneous oscillations, in contrast to the reduction of KA-induced gamma oscillations (vehicle 71.5±19.2 µV^2^, Y–27632 215.8±51.8 µV^2^, *p* = 0.005; Fig 9b). In *Ophn1* ^+/y^ slices, Y–27632 did not affect spontaneous oscillations (vehicle 137.5±20.9 µV^2^, Y–27632 180.8±40.9 µV^2^, *p* = 0.32; Fig 9b). The different effect of Y–27632 on spontaneous and KA-induced gamma oscillations was intriguing, but it may suggest that distinct network mechanisms generate spontaneous and KA-induced gamma oscillations [16].

## Discussion

The pathophysiology of intellectual disability is poorly understood, but mutations in single genes that result in cognitive deficits provide unique opportunities to discover the underlying mechanisms. Increasing evidence links synaptic malfunction to cognitive deficits [2], [27]. Mutation of the *OPHN1* gene in humans produces a non-specific X-linked mental retardation [19]. Reduced expression of oligophrenin-1, the protein encoded by *Ophn1*, results in altered dendritic spine morphology [20], [26] and learning impairments in mice [20]. Oligophrenin-1 has been previously shown to modulate endocytosis [22], [28], which alters responses to repetitive stimuli in excitatory synapses [23]. In contrast, its role in inhibitory terminals is less clear. In dentate gyrus granule neurons, it functioned as a regulator of synaptic vesicle availability [4], whereas in cultured CA1 neurons, oligophrenin-1 expression did not affect inhibitory transmission [28]. To further understand the role of oligophrenin-1 in cognitive deficits, we examined synchronous neuronal activity in the gamma frequency band. Reduced neuronal synchrony in the gamma range has been reported for various pathological conditions including Alzheimer's disease [29], ageing [30], schizophrenia [31] and autism [13]. Abnormal EEG recordings have long been associated with intellectual disabilities in humans [32], although until recently the link between mouse models and human disease was lacking [14]. Here we show that gamma oscillations are reduced in *Ophn1* ^−/y^ mice and propose that this is a result of the reduction in synaptic strength which arises from deficits in vesicle recycling.

The observation that both KA-induced and spontaneous gamma oscillations are reduced in *Ophn1* ^−/y^ slices is not surprising given the dependence of both on inhibitory neurotransmission [16] and that gamma oscillations place a high demand on inhibitory transmission [33], [34]. Kainate, acting via presynaptic receptors [35], has been demonstrated to facilitate neurotransmitter release via an alteration of the RRP [36]. Therefore the smaller RRP reported in this study could indeed explain the reduced gamma oscillation. Previous studies have demonstrated that the strength of the inhibitory input to pyramidal neurons modulates the power of gamma oscillations without changing the dominant frequency [15], [25]. Therefore, the alteration in vesicle availability appears the most likely candidate to explain the decreased power. Other potential explanations include alterations in the intrinsic excitability, although this seems unlikely as the intrinsic properties and firing frequency of interneurons and pyramidal neurons were not affected by the loss of expression of oligophrenin-1 (data not shown). An alternative explanation for the reduced gamma oscillations is that it results from fewer synapses on *Ophn1* ^−/y^ neurons. The rapid reversal of all the synaptic deficits [4] and spontaneous gamma oscillations (this study) argues against changes in synaptic numbers because it is unlikely that the doubling in numbers of synapses required to explain the results can occur in the rapid timescale shown here. Furthermore, the difference in RRP measured at single synapses in both the CA3 (this study) and dentate gyrus [4] using minimal stimulation demonstrates a direct functional impairment of synaptic mechanisms in *Ophn1^−/y^* mice.

### Pharmacological reversal of gamma oscillations *in vitro*

Despite learning disabilities being common disorders (affecting 2–3% of the population), the most effective treatment options remain special-needs education and similar approaches. Recent research has suggested that intellectual disabilities may be treatable with pharmacotherapy [37]. The cognitive deficits reported for *Ophn1* ^−/y^ mice have been proposed to result from over-activation of the RhoA signalling pathways [20]. Inhibition of the major downstream effector ROCK abolishes presynaptic changes in vesicle availability [4], postsynaptic alterations in synaptic plasticity [22], alterations in dendritic spine morphology [26] and more recently behavioural deficits [38]. In this study we have examined whether inhibition of ROCK activity could restore the altered gamma oscillations in *Ophn1* ^−/y^ mice. Pre-treatment with Y–27632 restored spontaneous gamma oscillations to wild type levels in *Ophn1*-/y slices, but was without effect on these oscillations in wild type slices. The reversal of spontaneous gamma presumably reflects the restoration of synaptic transmission towards normal levels. In contrast, Y-27632 reduced KA-induced gamma oscillations in both *Ophn1*-/y and *Ophn1*+/y slices, which was an unexpected observation given that it reverses the synaptic changes [4] and dendritic spine abnormalities [26] associated with reduced oligophrenin-1 expression. As the requirement for fast AMPA receptor-mediated synaptic transmission differs between KA-induced and spontaneous gamma oscillations [16], [39] the reversal of spontaneous, but not KA-induced oscillations may reflect selective reversal of excitatory transmission (Fig 4) with GABA-ergic transmission unaffected. The synaptic phenotype of impaired vesicle dynamics for both excitatory and inhibitory neurotransmission in CA3 synapses is analogous to that described in the dentate gyrus synapses where full reversal of inhibitory transmission was achieved [4]. So while not directly tested here, it is unlikely that the differential effects of Y-27632 are due to selective reversal of excitatory neurotransmission.

The neuronal networks that underlie the generation of spontaneous and KA-induced oscillations have been demonstrated to be dependent on different subpopulations of interneurons [16], [34], [40]–[42]. In KA-induced gamma oscillations, electrotonically-coupled parvalbumin-expressing basket cells are responsible for the phasic inhibition that synchronises pyramidal cell firing [11], [43], although multiple interneuron subtypes are active during gamma oscillations [34]. Spontaneous oscillations are also dependent on phasic inhibition, however current source density analysis suggests that s. lucidum interneurons are responsible, with ‘mossy fibre associated’ interneurons being the most likely candidate [16]. It is therefore possible that the differential effect of Y-27632 on KA-evoked and spontaneous gamma results from ROCK exerting different control of synaptic vesicle dynamics in the different interneuron populations, although this remains to be established.

Given that ROCK inhibition affected KA-induced oscillations to a similar extent in both wild-type and knockout slices, it is possible that the methodology used to generate gamma oscillations determines the effect of Y-27632. This has been demonstrated for the NR2B-selective NMDA receptor antagonist Ro25-6981 which potentiates KA-induced [14], but reduces tetanus-evoked gamma oscillations [44]. It would be interesting to examine the effects of inhibition of ROCK on gamma oscillations generated by activation of muscarinic receptors, as these are thought to be most similar to spontaneous oscillations [16].

Inhibition of ROCK may have non-specific effects on the network responsible for generation of KA-induced oscillations. For example, KA-induced oscillations are dependent on electrotonic coupling [45], [46] which may be altered by inhibition of ROCK [47]. Additionally, Y-27632 has been shown to modulate various ionic conductances such as Nav1.5 [48] and alter K^+^-induced contraction of smooth muscle [49], suggesting off-target effects which may specifically alter KA-induced oscillations. Therefore further studies are required to establish the underlying cause of the divergent effects of ROCK inhibition.

In this study we have examined whether inhibition of ROCK activity could restore the altered gamma oscillations in *Ophn1* ^−/y^ mice. A recent report demonstrated that peripheral application of ROCK inhibitors can improve spatial and working memory in rodents [50], raising the possibility that the use of ROCK inhibitors may provide a suitable pharmacotherapy for the reversal of cognitive deficits in this intellectual disability.
